# Reading the m^6^A-encoded epitranscriptomic information in development and diseases

**DOI:** 10.1186/s13578-024-01293-7

**Published:** 2024-09-28

**Authors:** Yunbing Chen, Ziyu Zhou, Yanxi Chen, Di Chen

**Affiliations:** 1 Center for Reproductive Medicine of The Second Affiliated Hospital, Center for Regeneration and Cell Therapy of Zhejiang University-University of Edinburgh Institute (ZJU-UoE Institute), Zhejiang University School of Medicine, Zhejiang University, Hangzhou, Zhejiang, 310003 China; 2 State Key Laboratory of Biobased Transportation Fuel Technology, Haining, Zhejiang 314400 China

**Keywords:** m^6^A, m^6^A readers, Development, Diseases, Cancers

## Abstract

*N*^6^-methyladenosine (m^6^A) represents the most prevalent internal and reversible modification on RNAs. Different cell types display their unique m^6^A profiles, which are determined by the functions of m^6^A writers and erasers. M^6^A modifications lead to different outcomes such as decay, stabilization, or transport of the RNAs. The m^6^A-encoded epigenetic information is interpreted by m^6^A readers and their interacting proteins. M^6^A readers are essential for different biological processes, and the defects in m^6^A readers have been discovered in diverse diseases. Here, we review the latest advances in the roles of m^6^A readers in development and diseases. These recent studies not only highlight the importance of m^6^A readers in regulating cell fate transitions, but also point to the potential application of drugs targeting m^6^A readers in diseases.

## Introduction

Epitranscriptomic modifications, encompassing chemical alterations of RNA molecules, play key roles in the regulation of most biological processes. RNA epigenetics, a significant branch of epigenetics, examines dynamic post-transcriptional RNA modifications, such as *N*^6^-methyladenosine (m^6^A). These modifications fine-tune RNA structures and functions, thereby influencing gene expression and regulation [1, 2]. Among the diverse repertoires of RNA modifications, m^6^A has emerged as a prominent and extensively investigated modification, distinguished by its remarkable prevalence as one of the most abundant modifications observed in eukaryotic mRNAs [3].

M^6^A modifications in mRNAs have been reported since the 1970s, while their key functions were not recognized until a deeper investigation into m^6^A-related enzymes was conducted [4]. In 2011, the Fat mass and obesity-associated protein (FTO) was identified as the first m^6^A demethylase, which catalyzes m^6^A RNA methylation to adenosine, making m^6^A a reversible modification under physiological and cellular regulation [5]. Since then, substantial studies have been conducted on the potential of this reversible m^6^A modification in different organisms or biological processes, shedding light on the roles of m^6^A-related proteins, including writers, erasers, and readers, in mediating m^6^A methylation, demethylation, and recognition for downstream regulation. Methyltransferases (writers), such as Methyltransferase-like 3 (METTL3) and METTL14, are responsible for installing m^6^A marks on RNA transcripts [6, 7]. Demethylases (erasers), including AlkB homolog 5 protein (ALKBH5) and FTO, mediate the removal of m^6^A modifications [5, 8]. Furthermore, m^6^A modifications are recognized by reader proteins, such as YTH domain family proteins (YTHDF1, YTHDF2, YTHDF3), YTH domain-containing family proteins (YTHDC1, YTHDC2), and the insulin-like growth factor 2 mRNA binding protein (IGF2BP) family proteins (IGF2BP1, IGF2BP2, IGF2BP3), to trigger downstream effects on m^6^A-modified transcripts [9]. M^6^A modifications are involved in the processes of RNA splicing, maturation, stability, localization, and translation, which in turn determine the fate of the modified RNAs in regulating cell fate [10–12].

Different cell types exhibit unique m^6^A profiles, shaped by the interplay of writers and erasers. The interpretation of m^6^A-mediated epigenetic information and the initiation of downstream processes are determined by m^6^A readers and their interacting proteins. These readers play a crucial role in recognizing and binding to m^6^A-modified RNA, setting off a cascade of events that influence gene expression, cellular differentiation, and various physiological responses. Essentially, the dynamic interaction between m^6^A readers and their interacting proteins serves as a key regulatory mechanism in transmitting epigenetic information into functional cellular outcomes. In this review, we focus on the m^6^A readers, especially how different readers play key roles in different biological processes, and how the defects in m^6^A recognition contribute to diseases.

## The machinery for m^6^A modification

Epigenetic modifications, including DNA methylation, RNA modification, histone modification, chromatin remodeling, and noncoding RNA regulation, play particularly crucial roles in most if not all biological processes. Since it was first discovered in the 1970s, m^6^A has been discovered to be the most prevalent internal modification present in the mRNAs of all higher eukaryotes [13]. Advancements in high-throughput sequencing techniques have revolutionized the study of m^6^A modifications, extending their understanding from prokaryotic bacteria to eukaryotic organisms including humans. In 2012, the comprehensive profiling of m^6^A in mammalian cells for the entire transcriptome was achieved through the development of m^6^A antibody-based RNA-immunoprecipitation strategies, such as m^6^A-seq and Methylated RNA immunoprecipitation sequencing (MeRIP-seq) [14, 15]. These innovative techniques allowed researchers to identify and study the distribution of m^6^A modifications across RNA molecules. Interestingly, m^6^A was found to be predominantly enriched in regions such as the 3′ untranslated regions (3′UTRs) and in proximity to stop codons, a feature that is highly conserved in different species [16]. M^6^A modifications have been identified in different RNA types, such as messenger RNA (mRNA), transfer RNA (tRNA), ribosomal RNA (rRNA), circular RNA (circRNA), microRNA (miRNA), and long noncoding RNA (lncRNA). Each mRNA molecule, on average, contains three to five m^6^A modifications, which constitute approximately one-third of the total mammalian mRNAs [17].

**Fig. 1:**
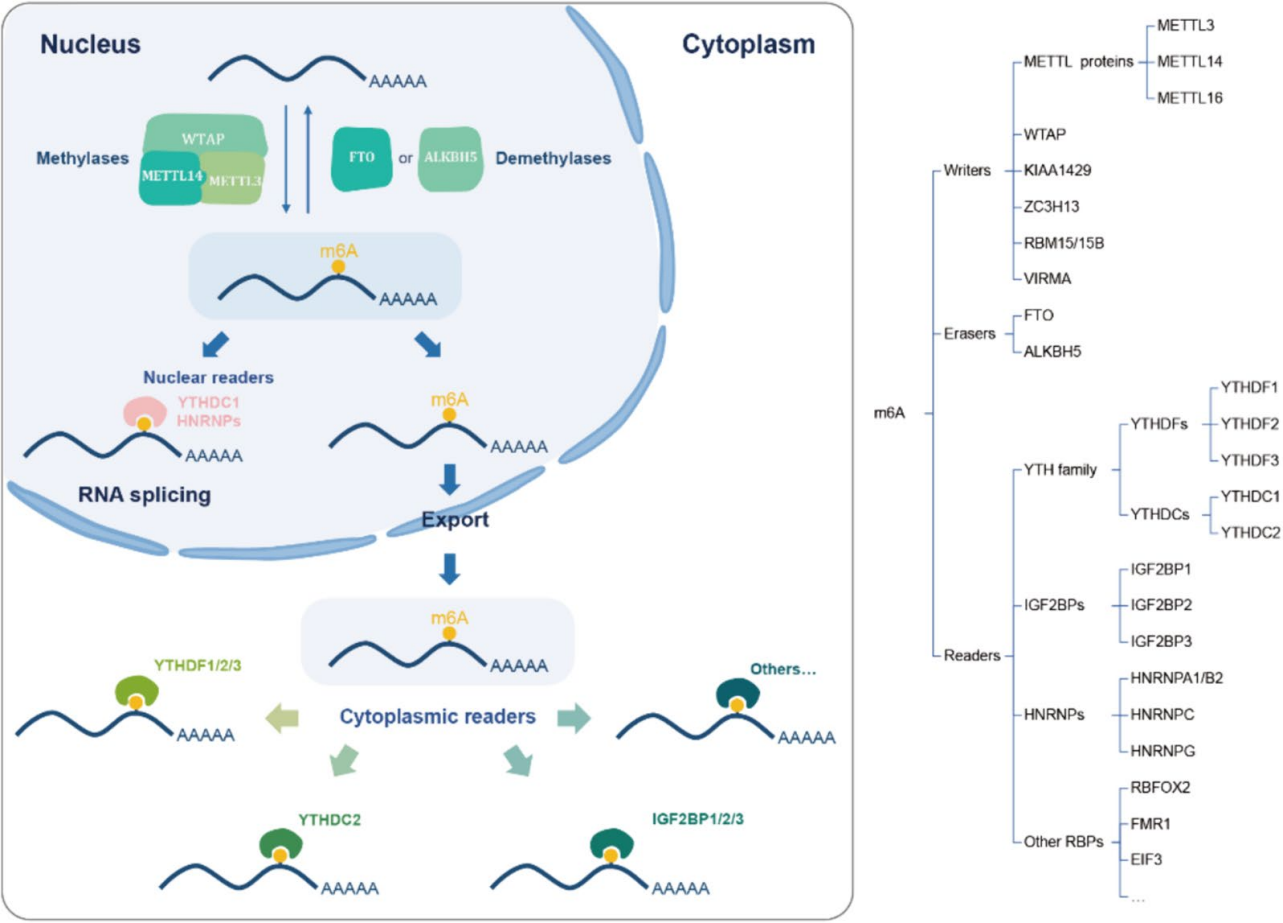
Dynamic m^6^A modification in the nucleus and cytoplasm. The m^6^A modification exhibits dynamic processes in cellular compartments, with distinct mechanisms operating in the nucleus and cytoplasm. In the nucleus, the methyltransferase complex (WTAP, METTL3/METTL14) catalyzes m^6^A addition to RNAs, while nuclear demethylases (FTO or ALKBH5) facilitate m^6^A removal. Nuclear reader proteins, such as YTHDC1 and HNRNPs, recognize m^6^A sites and influence RNA splicing. Subsequently, m^6^A-modified RNAs are transported to the cytoplasm, where cytoplasmic reader proteins (e.g., YTHDF1/2/3, YTHDC2) further bind to regulate it. Additional proteins, such as IGF2BP1/2/3, are also involved in RNA recognition and regulation

The understanding of the reader, writer, and eraser is gradually integrated to form an understanding of the overall role of m^6^A in the mRNA's life cycle (Fig. 1). The m^6^A modification is installed co-transcriptionally by a methyltransferase complex (MTC) consisting of the METTL3 catalytic component and other accessory subunits such as METTL14, Wilms Tumor 1-Associating Protein (WTAP), Vir-like m6A methyltransferase associated (VIRMA), and Zinc finger CCCH-Type containing 13 (ZC3H13) [18]. This writer complex binds to the mRNAs and transfers the methyl group to the specific adenosines, which usually contain the RRACH motifs [15]. METTL3, METTL14, and METTL16 are the key writers in the m^6^A pathway. METTL3 has been implicated in various biological processes, including modulation of alternative splicing, stabilization of target mRNAs, regulation of hematopoietic stem cell self-renewal, and involvement in tumorigenesis [7, 19]. METTL14 forms a heterodimer with METTL3, which serves as the catalytic core of the m^6^A RNA methyltransferase complex. METTL14 enhances the catalytic activity of METTL3 and contributes to RNA binding and substrate specificity, thus stabilizing the complex and facilitating its function [7, 10, 20]. METTL16, on the other hand, is involved in methylating specific mRNAs, lncRNAs, and U6 small nuclear RNAs (snRNAs), as well as regulating S-adenosylmethionine (SAM) homeostasis [6, 21, 22].

M^6^A erasers, also known as demethylases, are involved in shaping the m^6^A epitranscriptome by converting m^6^A into adenosine, making m^6^A modification a reversible, dynamic, and regulable epigenetic process [5]. These erasers play critical roles in the dynamic and signal-dependent removal of m^6^A, thereby influencing its function. Among the identified demethylases, the FTO gene was the first discovered m^6^A demethylase and was initially associated with an increased risk of obesity [5]. Another demethylase, ALKBH5, predominantly localizes in the nuclear speckles. Through its demethylation activity, ALKBH5 modulates nuclear RNA export and RNA metabolism, ultimately regulating gene expression [8]. Together, the m^6^A writers and erasers function cooperatively to determine the m^6^A landscapes in different cell types, and mediate the transcriptomic change during cell fate transition (Fig. 1).

Although determined by m^6^A writers and erasers, how the m^6^A modifications contribute to the mRNA metabolism and cell fate transition is mediated by m^6^A readers, which bind to m^6^A-modified RNAs and recruit interacting proteins to regulate RNA metabolism. Our understanding of m^6^A readers and their associated functions is undergoing rapid and profound advancements. An escalating body of research reveals that these novel m^6^A readers exert distinct functionalities in various cellular contexts and model systems. Fundamentally, m^6^A readers execute their roles by interacting with m^6^A-modified mRNA substrates [18]. Dysregulation of different m^6^A readers has been intensively studied in diverse disease conditions [23–25]. Resolving the functions of the m^6^A readers is crucial for understanding the pathogenesis of diseases and exploring potential diagnostic approaches.

## The structure and function of m^6^A readers

**Fig. 2:**
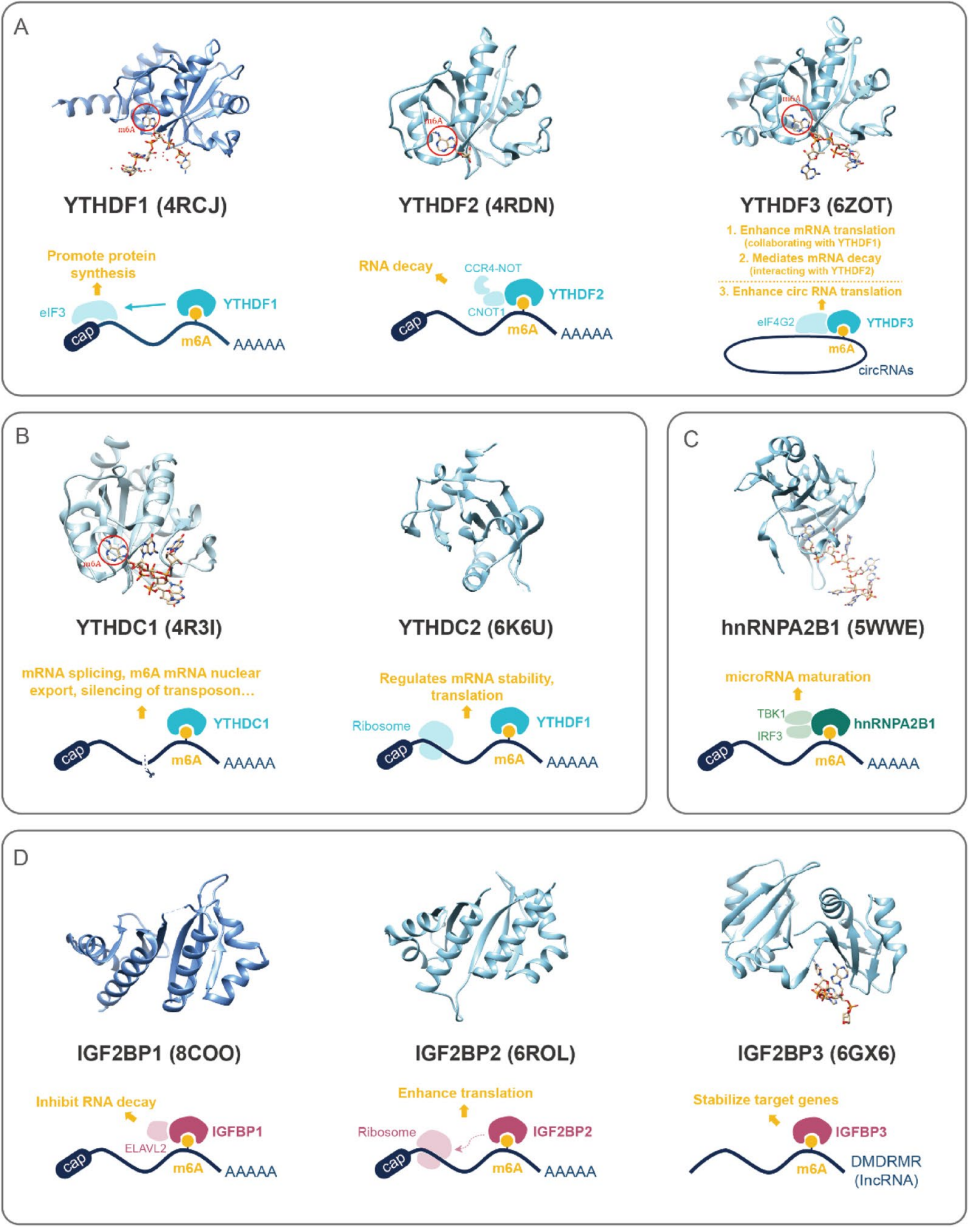
Structural Insights into m^6^A Reader Proteins and Their Functions in RNA Regulation. The illustration comprehensively describes the 3D structures and functions of distinct families of m^6^A reader proteins. A. YTHDF family, comprising YTHDF1, YTHDF2, and YTHDF3, serves as pivotal readers for m^6^A-modified RNAs. YTHDF1 facilitates RNA translation by recognizing m^6^A modification and engaging with eIF3. Meanwhile, YTHDF2 promotes the degradation of target RNAs by recognizing m^6^A modification and interacting with the carbon catabolite repressor 4-negative on TATA (CCR4-NOT) complex. YTHDF3 collaborates with YTHDF1 to enhance mRNA translation as well as mediates mRNA decay through interaction with YTHDF2. It can also enhance circRNA translation, by interacting with eIF4G2. B. YTHDC family consists of YTHDC1 and YTHDC2. YTHDC1 is involved in mRNA splicing, m^6^A mRNA nuclear export, and silencing of transposons, while YTHDC2 exerts influence over the stability and translation of specific target genes. C. hnRNPA2B1 recognizes and binds to m^6^A sites, exerting regulatory effects on RNA processing and metabolism through interacting with TBK1 and IRF3. D. IGF2BP family includes IGF2BP1, IGF2BP2, and IGF2BP3. IGF2BP1 prevents degradation of target RNAs by interacting with ELAVL2, while IGF2BP2 enhances translation efficiency by modulating ribosome binding to target mRNAs. IGF2BP3 stabilizes m^6^A-enriched target RNAs, safeguarding them from degradation

Among all the m^6^A readers, the most extensively studied are members of the YTH family and IGF2BP family, which separately utilize YTH and KH domains to specifically bind to m^6^A sites. This binding facilitates the recruitment of various protein complexes, which play pivotal roles in pre-mRNA splicing, mRNA degradation, translation, and nuclear transport processes. In addition to the above two families of proteins, there are other readers, including the heterogeneous nuclear ribonucleoproteins (HNRNP) family and other RNA-binding proteins (RBPs). The interaction between these m^6^A readers and their respective binding sites is crucial for the modulation of gene expression and the control of diverse cellular functions (Fig. 2).

### YTH family of m^6^A readers

YTH family members feature a highly conserved YTH domain at their C-terminus, essential for specific recognition of m^6^A-modified mRNAs. This domain, through several conserved aromatic amino acids, forms a unique cage-like structure capable of precisely accommodating the methylated adenosine. Recognition of methylated adenosine by the YTH domain is facilitated by π-π interactions between the purine base and benzene ring, and cation-π interactions between the methyl group and aromatic amino acids. On the contrary, the absence of methylation on adenosine weakens the interaction between the YTH domain and m^6^A-modified RNAs [9, 26, 27]. Moreover, certain amino acids within the YTH domain can interact with other bases on the RNA (Fig. 2A-B). For instance, when methylated adenosine is at the 0 position, YTHDC1 shows a preference for guanine instead of adenine at the -1 position, which is attributed to the ability of guanine to establish hydrogen bonds with the V382 residue of the YTH domain in the YTHDC1 [28]. Structural analyses have revealed the intricate molecular mechanisms by which the YTH domain-containing m^6^A readers recognize m^6^A-modified RNA.

Proteins containing the YTH domain, based on their structural similarities, can be further classified into three main categories: the YTHDF family proteins, YTHDC1, and YTHDC2. The YTHDF family consists of three highly homologous proteins: YTHDF1, YTHDF2, and YTHDF3 (Fig. 2A). Each protein is characterized by a C-terminal YTH domain and an N-terminal domain enriched with proline, glutamine, and asparagine residues [29]. These proteins are integral to the translation and degradation of m^6^A-modified mRNAs within the cytoplasm. YTHDF2, the first reader protein discovered in 2014, has garnered significant attention. Its C-terminal domain selectively binds to m^6^A-containing mRNAs, while its N-terminal domain directly interacts with the SH domain of the CNOT1 subunit [13]. This interaction is responsible for recruiting subunits to form the CCR4-NOT complex, which leads to the de-adenylation and subsequent degradation of the bound m^6^A-modified mRNAs [30, 31]. In contrast, the primary functions of YTHDF1 and YTHDF3 are related to translation. YTHDF1 actively promotes protein synthesis through interactions with Eukaryotic translation Initiation Factor 3A (eIF3A) and eIF3B, as well as both 40S and 80S ribosome subunits [12]. YTHDF3 plays a pivotal role in regulating the fate of m^6^A-modified mRNA, as it collaborates with YTHDF1 to enhance mRNA translation and mediates mRNA decay through interaction with YTHDF2. Additionally, YTHDF3 recruits eIF4G2 to m^6^A sites, initiating the translation of circRNAs [32–34] (Fig. 2A).

YTHDC1 is one of the m^6^A readers which localized in the nucleus [35] (Fig. 1). As the first reader whose specific structure was identified, it undertakes diverse m^6^A-associated functions, including modulating mRNA splicing, mediating m^6^A-modified mRNA nuclear export, and participating in the silencing of transposon [36–39]. Notably, YTHDC2 stands out with distinct features that set it apart from other YTH family proteins. Enriched in testes and function in both the cytoplasm and nucleus, YTHDC2 primarily regulates mRNA stability and translation [40, 41]. Despite its YTH domain showing a relatively weaker m^6^A-binding affinity compared to its counterparts, UV Cross-Linking and Immunoprecipitation (CLIP) analysis of YTHDC2 binding sites across the transcriptome suggest a low overlap with m^6^A sites in mRNAs, hinting that YTHDC2 may recognize specific m^6^A sites or employ alternate binding mechanisms to modulate m^6^A-modified mRNAs [42]. Apart from the YTH domain, YTHDC2 also harbors other RNA-binding domains, such as the R3H domain, which are thought to bridge interactions between m^6^A-containing mRNAs and ribosomes, thereby fostering effective translation [40] (Fig. 2B).

In conclusion, the structure of the YTH domain family proteins, characterized by their conserved YTH domain and specific aromatic amino acids, is key to their different m^6^A-dependent regulation. This structural configuration allows for a variety of interactions with RNAs, facilitating their diverse functions in RNA metabolism, including translation, degradation, and splicing. Their distinct structural design is fundamental for their essential roles in m^6^A-related cellular processes.

### IGF2BP family of m^6^A readers

The IGF2BPs have been recognized as a novel class of m^6^A readers in recent years. These proteins predominantly form granular messenger ribonucleoprotein (mRNP) structures within the perinuclear region of the cytoplasm, thereby influencing the fate of mRNAs [41]. Among them, IGF2BP1, IGF2BP2, and IGF2BP3 can recognize and bind to m^6^A-modified RNA (Fig. 2D). These three IGF2BP proteins exhibit considerable structural conservation, with an overall amino acid sequence homology of about 56%. Remarkably, IGF2BP1 and IGF2BP3 share up to about 73% of amino acid sequence homology [43]. Their protein domains are similarly organized in terms of order and spacing, each featuring two RNA-recognition motifs (RRMs) at the N-terminus and four KH domains at the C-terminus [44]. The KH domains are primarily responsible for RNA binding, while the RRM domains contribute to the stabilization of IGF2BP-RNA complexes, with a half-life exceeding 2 h [45, 46]. It is predominantly through the third and fourth KH domains (KH3-4) that the IGF2BPs recognize m^6^A sites and specifically bind to the target RNAs. Functionally, IGF2BPs play pivotal roles in the post-transcriptional regulation of RNAs, including translational control, RNA stability, and RNA localization in neuronal cells [11, 46, 47] (Fig. 2D).

### HNRNP family as m^6^A readers

HNRNPs comprise a diverse ensemble of RNA-binding proteins, encompassing approximately 20 unique proteins ubiquitously expressed in human tissues [48]. Recent studies have identified several HNRNPs, including HNRNPA2B1, HNRNPC, and HNRNPG, which are either directly or indirectly involved with m^6^A modifications. Structurally, these proteins harbor RNA recognition motifs (RRMs), typically consisting of 80–90 amino acids to form a sandwich-like structure with two α-helices against a four-stranded β-sheet. Additionally, some HNRNPs are characterized by an RGG box, a domain composed of repeated arginine and glycine residues. This domain enhances their association with specific RNA sequences or modifications including m^6^A [49]. By far, only the RRMs in HNRNPA2B1 have been experimentally demonstrated to directly and specifically interact with m^6^A-modified RNA (Fig. 2C). These interactions subsequently modulate various RNA metabolic processes, such as RNA splicing [50].

Contrarily, HNRNPC, a well-characterized RNA-binding protein with an affinity for U-rich sequences, recognizes m^6^A indirectly. Its recognition is contingent on discerning alterations in RNA structure induced by m^6^A modifications [51]. Specifically, the presence of m^6^A at certain sites can induce structural rearrangements of RNAs, uncovering U-rich sites that were previously masked. These exposed sites can be recognized by HNRNPC, enabling its subsequent interaction with these m^6^A-modified RNAs [30]. Similar to HNRNPC, HNRNPG functions as an indirect reader, recruiting m^6^A reader YTHDF2 to decrease the stability of target mRNAs [50]. Intriguingly, while the RGG box substantially augments the binding affinity between the proteins and RNAs, its influence on the specific binding of HNRNPG to RNA is comparatively marginal [52].

### Other m^6^A readers

In addition to the previously mentioned protein families, other RBPs are also serving as m^6^A readers, recognizing the m^6^A modification recognition in an m^6^A sequence-dependent manner. RBFOX2, a recently discovered m^6^A reader with an RRM, specifically binds to m^6^A sites at the promoter region on mRNAs by recognizing the UGCAUG sequence. With this binding structural characteristic, RBFOX2 can increase chromatin accessibility in an m^6^A-dependent manner, further influencing RNA methylation levels and RNA abundance, as well as the level of H3K4me3 modification in chromatin [53]. Other RBPs, such as Fragile X Mental Retardation Protein 1 (FMR1) and eukaryotic initiation factors 3 (elF3), have been also identified as m^6^A readers [9, 26, 27]. However, their specific binding mechanisms and functions remain to be investigated.

These diverse families and members of readers indicate that m^6^A modifications should be recognized by different readers and associated protein complexes to trigger different downstream effects on the m^6^A-modified RNAs, which in turn, participate in the regulation of diverse developmental processes such as embryogenesis, hematopoiesis, neurogenesis, gametogenesis, and others.

## The roles of m^6^A readers in development

### M^6^A readers in embryogenesis

Embryogenesis is a highly orchestrated process by which a zygote develops into an embryo. In this complex biological process, gene expression is precisely controlled and coordinated at transcriptional and post-transcriptional levels to achieve different cell fates through differentiation. An accumulation of studies highlights the importance of m^6^A readers in regulating embryonic development.

The development of embryos is initially directed by maternal-derived gene products, followed by maternal-to-zygotic transition (MZT) to gradually remove maternal materials and activate the zygotic genome for expression. M^6^A readers have been discovered to play key roles in the clearance of maternal mRNAs during embryogenesis. Specifically, YTHDF2 facilitates the degradation of a subset of m^6^A-modified maternal mRNAs, participating in the first wave of RNA degradation during MZT [54, 55]. Conversely, YTHDC1 protects certain m^6^A-modified maternal mRNAs from degradation and promotes their translation, providing necessary proteins and signals for Zygotic Genome Activation (ZGA). Notably, YTHDC1 stabilizes the mRNAs of Y-Box Binding Protein 3 (YBX3), a transcription factor that activates crucial zygotic genes, including Nanog Homeobox (NANOG), SRY-Box Transcription Factor 2 (SOX2), and POU Class 5 Homeobox 1 (POU5F1). Therefore, through modulating the stability and translation of m^6^A-modified mRNAs, m^6^A readers intricately shape MZT dynamics and efficacy [56].

Embryonic stem cells (ESCs) are the in vitro counterparts of developing pluripotent embryonic cells, serving as the cellular systems to study the regulatory mechanisms governing stem cell pluripotency and differentiation. YTHDF1 and YTHDF3 have emerged as crucial regulators of mouse embryonic stem cell (mESC) pluripotency, germ layer formation, and cardiomyocyte (CM) differentiation [57]. Specifically, depletion of YTHDF1 in mESCs leads to enhanced stemness, characterized by elevated expression levels of pluripotency-associated genes such as Developmental Pluripotency Associated 3 (DPPA3) and Kruppel-Like Factor 2 (KLF2) [57]. Conversely, the knock-down of YTHDF3 in ESCs results in the upregulation of genes involved in germ layer formation, indicative of the loss of pluripotency phenotype. Furthermore, YTHDF1 depletion significantly impairs CM differentiation, marked by decreased expression of CM-specific genes, while depletion of YTHDF3 promotes in vitro cardiac myogenesis by enhancing the expression of CM-specific genes [57].

YTHDF1 has been revealed to play a vital role in modulating the pluripotency of porcine-induced pluripotent stem cells (piPSCs). The deficiency of m^6^A in piPSCs negatively impacts self-renewal ability and enhances the initiation of the differentiation process in an m^6^A reader-dependent manner [58, 59]. This effect is mechanistically attributed to the inhibition of YTHDF1-mediated Janus Kinase 2 (JAK2) translation and the concurrent disruption of YTHDF2-dependent decay of Suppressor of Cytokine Signaling 3 (SOCS3) mRNA. Consequently, these molecular modulations culminate in the suppression of JAK2-STAT3 pathway activation and subsequent transcriptional downregulation of KLF4 and SOX2, both of which are pivotal regulators of pluripotency [58]. Additionally, recent studies have revealed the significant roles of YTHDF1 in regulating the self-renewal capacity of mouse female germline stem cells (mFGSCs). Specifically, m^6^A binding protein YTHDF1 is found to be instrumental in modulating the self-renewal processes of mFGSCs [59].

YTHDC1 is essential for the self-renewal and differentiation of mESCs by binding to m^6^A-modified mRNAs. It is necessary for proper rRNA synthesis and repression of the 2-cell (2C) transcriptional program in mESCs, which resembles regulation by the Long Interspersed Nuclear Element 1 (LINE1) scaffold. YTHDC1 recognizes m^6^A on LINE1 RNAs in the nucleus, regulating LINE1-Nucleolin (NCL) partnership, KRAB-associated protein 1 (KAP1) chromatin recruitment, and H3K9me3 establishment on 2C-related retrotransposons. YTHDF1 depletion disrupts these processes, leading to increased transcriptional activities [60]. Taken together, m^6^A readers are important players in regulating embryonic development.

### M^6^A readers in hematopoiesis and immunocytogenesis

The development of the hematopoietic system commences during the early embryonic stages, marked by the first appearance of hematopoietic stem cells (HSCs) within the dorsal aorta. These HSCs originate from a specialized subset of endothelial cells, termed hematopoietic endothelial cells, which undergo the endothelial-hematopoietic transition (EHT) process for differentiation [61]. Following EHT, these HSCs migrate to the fetal liver, where they undergo proliferation, and further relocate to the bone marrow for differentiating into various precursor cells based on specific functional requirements, such as granulocyte-megakaryocyte progenitors (GMPs) and lymphoid-primed multipotent progenitors (LMPPs). These precursor cells further differentiate into mature blood and immune cells, which migrate to their designated lifelong hematopoietic sites for respective functions [61].

Recent studies have highlighted the pivotal role of m^6^A modification in hematopoietic development, with a particular focus on YTHDF2, a key m^6^A reader holding an indispensable position in both HSCs and their subsequent cellular fates [62]. In HSCs, the depletion of YTHDF2 impedes m^6^A-dependent mRNA decay, leading to alterations in the Wnt signaling pathway. This modulation enhances the proliferation of functional HSCs, which could be observed in both murine HSCs (mHSCs) and human umbilical cord blood HSCs [62]. Furthermore, YTHDF2 acts as an inhibitor of the HSC inflammatory pathway, preserving HSC functionality via m^6^A-mediated regulation. In YTHDF2-deficient HSC mouse models, young mice demonstrated increased levels of multiple m^6^A-modified inflammatory transcripts, while older mice faced difficulties in multilineage hematopoiesis [62].

At the later stage of cellular development, YTHDF2 plays a pivotal role in influencing the cell fate decisions of innate and adaptive immune cells [63, 64]. Within B cells, YTHDF2 emerges as a key regulator of IL-7-induced B-cell progenitor proliferation. Its role in mediating mRNA decay is vital for the subsequent development and differentiation of B-cell progenitors. With the absence of METTL14, the binding of YTHDF2 to its target RNA diminishes, resulting in significant inhibition of pro-B cell proliferation, an altered transition from large-pre-B to small-pre-B cells, and notable abnormalities in gene expression programs essential for B cell development [63]. In tumor-associated macrophages (TAMs), YTHDF2 is instrumental in modulating their anti-tumor immune capabilities. Specifically, YTHDF2, by interacting with RBM4, facilitates the degradation of m^6^A-modified mRNAs of Signal Transducer and Activator of Transcription 1 (STAT1), thereby inhibiting the polarization of M1 TAMs, consequently diminishing both the innate and adaptive anti-tumor immunity of TAMs [64].

In addition to YTHDF2, other m^6^A readers have been identified to have an intricate influence on hematopoietic development. IGF2BP2 demonstrates high expression in long-term HSCs (LT-HSCs) and serves as a pivotal factor in preserving the function of HSCs. This elevated expression aids in maintaining the stability of its downstream target, BMI1 Proto-Oncogene, Polycomb Ring Finger (BMI1) RNAs. As a result, the suppressive effect of BMI1 on the expression of mitochondrial-associated genes is ensured [65]. Consequently, this controls mitochondrial activity, acting as a safeguard against the degradation of HSC functionality [65]. Experimental evidence from studies on IGF2BP2 knock-out mice has further underscored its critical role in hematopoietic processes. These mice exhibited a notably reduced LT-HSC number, increased apoptosis rates, and severely compromised long-term hematopoietic regeneration capabilities, further underlining the indispensability of IGF2BP2 in maintaining HSC function [65]. Together, m^6^A readers play key roles in regulating hematopoiesis.

### M^6^A readers in neurogenesis

Neuronal development involves a series of events, from generating neural progenitor cells to differentiating them into specialized neurons. This dynamic process centers on neural stem cell development and occurs during both embryonic development and throughout an organism's life [66]. Key to this intricate process is the role of m^6^A readers, influencing neural stem cell fate decisions by regulating target mRNAs.

The deficiency of YTHDF2 exerts profound effects on cortical development, neurogenesis, and gliogenesis [67]. YTHDF2 deficiency disrupts the proliferative capacity and cell cycle progression of neural progenitor cells, thereby perturbing their symmetric and asymmetric divisions, ultimately leading to aberrant neurite outgrowth and morphological abnormalities in neurons [67]. In YTHDF2-deficient mice, an array of abnormal brain developmental features manifests, including decreased cortical thickness and reduced neural stem/progenitor cells (NSPCs) proliferation. The diminished cortical thickness primarily arises from a significantly thinner cortical fiber layer and periventricular zone, which are composed of neurons (Dcx +) and basal progenitor cells (Tbr2 +) [67]. Notably, YTHDF2-deficient NSPCs retain their ability to undergo neuronal differentiation in vitro; however, their capacity to differentiate into glial cells becomes impaired, indicating a bias towards neuronal lineage differentiation [67]. Mechanistically, this biased differentiation may arise from the upregulation of m^6^A-modified mRNAs such as Neuropilin 2 (NRP2), Neurexin 3 (NRXN3), Fibronectin Leucine Rich Transmembrane Protein 2 (FLRT2), Protein Tyrosine Phosphatase Receptor Type D (PTPRD), and Discoidin Domain Receptor Tyrosine Kinase 2 (DDR2), which involved in the negative regulation of neurodevelopment in YTHDF2-deficient NSPCs. Dysregulation of these m^6^A-modified transcripts likely disrupts the proper differentiation trajectory of NSPCs, contributing to the observed aberrant cellular phenotypes [67].

YTHDF1 has emerged as a critical factor in the regulation of hippocampus-dependent learning and memory processes in mice [68, 69]. YTHDF1 knock-out mice exhibit impairments in spatial learning and memory, the basal synaptic transmission of hippocampal CA1 neurons, and long-term potentiation (LTP). The underlying mechanisms behind these impairments can be attributed to a substantial reduction in the abundance of crucial proteins involved in LTP within the postsynaptic density (PSD). This decrease in protein levels is a direct consequence of YTHDF1 loss, indicating its pivotal role in promoting the translation of transcripts relevant to neuronal function [69]. Collectively, these findings highlight the central involvement of YTHDF1 in regulating the generation of hippocampus-dependent learning and memory, primarily through its capacity to enhance the translation of neuron-related transcripts.

Both YTHDF1 and YTHDF2 have been found in cerebellar granule cells and are thought to be strongly associated with granule cell growth and in vivo cerebellar synapse formation. Combined RIP-Seq results for YTHDF1 and YTHDF2 with quantitative proteomic analysis or RNA-seq following knock-down of these proteins revealed their role in regulating local translation of specific proteins. YTHDF1 controls the translation of Dishevelled Segment Polarity Protein 1 (DVL1), while YTHDF2 regulates Wnt Family Member 5A (Wnt5a). Both proteins are crucial for controlling axon growth in granule cells [70]. Furthermore, the knock-out of YTHDF1 or YTHDF2 in granule cells was found to significantly promote in vitro granule cell axon and parallel fiber growth, cerebellar synapse formation, and motor coordination [70].

IGF2BPs have been implicated in the intricate regulation of mRNA dynamics during neural development, especially the establishment of synaptic connections [71]. Among these proteins, IGF2BP1 stands out as a notable example, showcasing its ability to precisely control the spatial and temporal aspects of regulating local Actin Beta (ACTB) mRNAs. Functionally, IGF2BP1 sequesters ACTB mRNAs within cytoplasmic protein-RNA complexes, commonly referred to as mRNPs. These mRNPs are subsequently transported to dendrites or axons during the hippocampus development [71]. Notably, the phosphorylation of IGF2BP1 by SRC kinase facilitates the release of ACTB mRNAs, enabling its translation and local enrichment within specific subcellular compartments, and facilitating growth cone guidance, a fundamental process in neural development and synaptic connectivity [71]. Therefore, m^6^A readers are critical regulators for neurogenesis.

### M^6^A readers in reproductive system

Post-transcriptional regulation and utilization of the maternal transcriptome are crucial for meiotic maturation, fertilization, and early embryonic development [54]. Throughout the process of folliculogenesis, which marks the oocyte's growth stage, critical biomaterials and the maternal transcriptome are compiled to ensure oocyte competency [72]. Additionally, male reproductive cells progress through three distinct phases: mitotic division for spermatogonia self-renewal, meiotic divisions in spermatocytes, and the transformation of haploid spermatids into mature sperm via spermiogenesis [73]. Post-transcriptional mechanisms are essential for comprehending spermatogenesis since large dynamic changes in the transcriptome during mammalian spermatogenesis have been documented, along with the existence of uncoupled transcription and translation at specific stages of spermatogenesis [74].

YTHDF2, a cytoplasmic entity, is a consistent presence throughout the gametogenesis of mammals, where it is critical for the proper functioning of oocytes. Forced expression of YTHDF2 in somatic granulosa cells while selectively deleting it from growing oocytes in mice using a maternal conditional deletion strategy leads to female sterility [75]. YTHDF2 also plays a crucial role in spermatogenesis and fertility [74]. The male mice with germ cell-specific knock-out of YTHDF2 exhibited sterility, with their sperm displaying abnormalities, reduced motility, and impaired fertilization capacity. In normal germ cells, during the transition from differentiated spermatogonia to pachytene spermatocytes, a majority of m^6^A-modified RNA targets bound by YTHDF2 were degraded. However, in YTHDF2 conditional knock-out (cKO) mice, these targeted mRNAs persisted in pachytene spermatocytes, leading to delayed degradation. These persistent mRNAs were primarily associated with pathways involved in transcriptional regulation, consequently disturbing the transcriptome of both round spermatids and elongated spermatids [55]. Additionally, studies have demonstrated that Ythdf2 knock-out mice, despite undergoing normal spermatogenesis, develop a condition called oligoasthenozoospermia (OAT), characterized by reduced sperm count and motility, along with increased germ cell apoptosis [74]. The impact of YTHDF2 on spermatogenesis and fertility extends beyond mice. In zebrafish, it has been observed that homozygous mutant males (Ythdf2^−/−^), regardless of maternal genotype, produce on average approximately 70% of embryos that fail to progress beyond the one-cell stage, suggesting potential defects in sperm function [76, 77]. However, it has also been proposed that deletion of YTHDF2 in mice has a partially permissive effect, resulting in female-specific sterility with little effect on males [75].

YTHDC1 is crucial for the development of spermatogonia in males and the growth and maturation of oocytes in females [78–80]. In YTHDC1-deficient oocytes, maturation is halted at the primary follicle stage. Interestingly, the absence of YTHDC1 leads to significant changes in alternative polyadenylation, resulting in alterations in the length of the 3'UTR of mRNAs [81]. Additionally, YTHDC1 deficiency causes substantial defects in alternative splicing in oocytes. Most of these splicing defects can be rescued by introducing wild-type YTHDC1, but not a mutant version lacking m^6^A-binding ability. YTHDC1 interacts with key factors involved in pre-mRNA 3′ end processing, such as Cleavage and Polyadenylation Specific Factor 6 (CPSF6), Serine and Arginine Rich Splicing Factor 3 (SRSF3), and SRSF7. This highlights the critical role of YTHDC1 in pre-mRNA processing within the oocyte nucleus and suggests potential non-redundant functions throughout fetal development [81]. The specific inactivation of YTHDC1 using DDX4-Cre in the germline resulted in YTHDC1-cKO mice, which showed normal development but experienced a significant reduction in spermatogonia by postnatal day 8. By postnatal day 25 and adulthood, the testes of YTHDC1-cKO mice exhibited a complete absence of germ cells, leading to a Sertoli-cell-only phenotype, indicating the essential role of Ythdc1 in spermatogonia development and male fertility [81]. Altogether, m^6^A readers are essential factors for regulating gametogenesis for both males and females.

## The roles of m^6^A readers in cancers

**Fig. 3:**
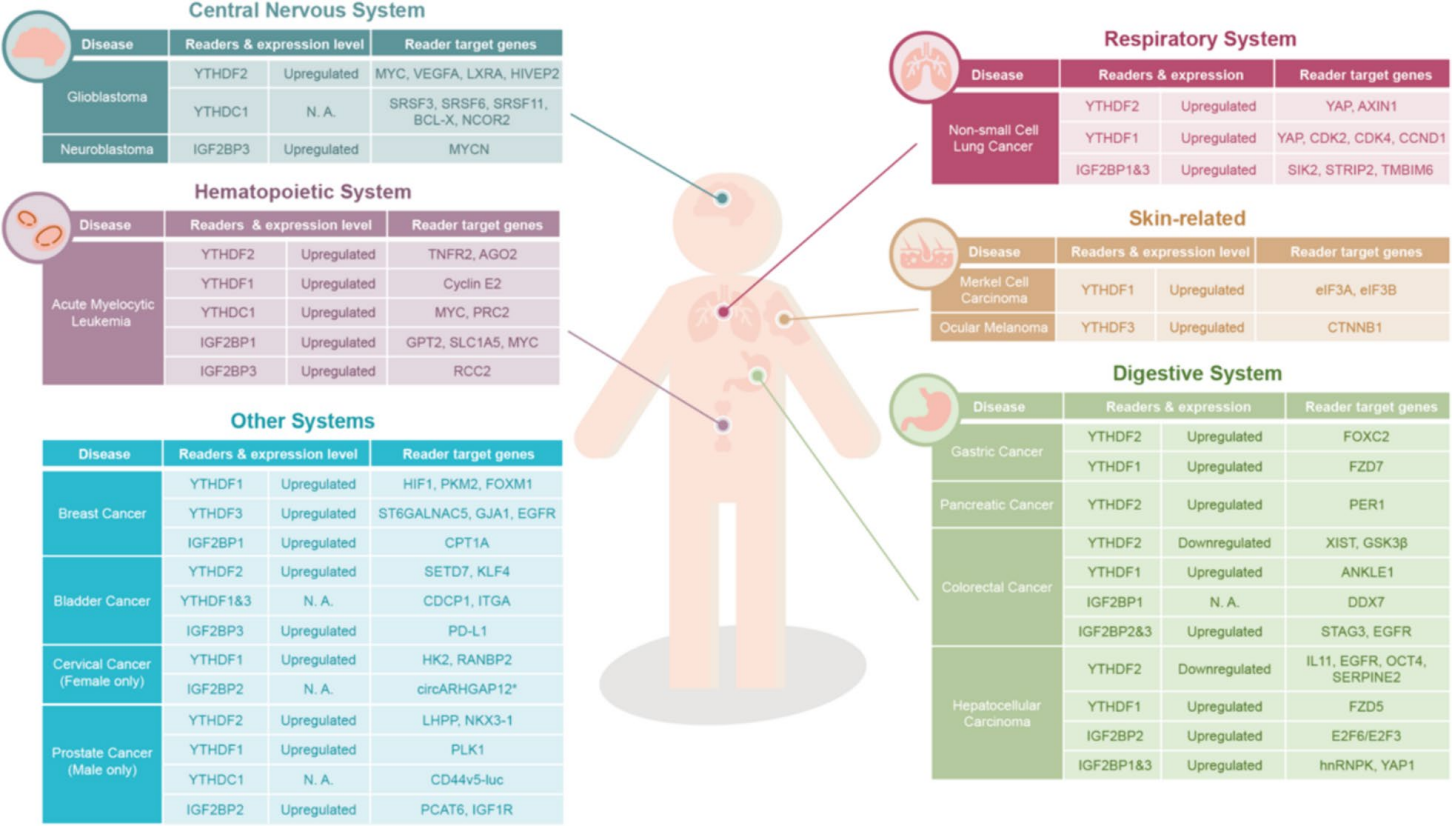
The relationship between different m^6^A reader families and associated cancers. Dysregulation of m^6^A readers is associated with cancers in various human systems, including the central nervous system, digestive system, respiratory system, urogenital system, and hematopoietic system. The expression levels of reader proteins in cancer conditions and the potential targets recognized by different m^6^A readers are indicated. The circARHGAP12, marked with an asterisk (*) in the figure, is a circRNA

The m^6^A modification not only influences the normal development process of stem cells but also regulates aberrant stem cell behaviors, such as those observed in cancers (Fig. 3) [25, 82]. Investigating the significance of m^6^A readers in cancers becomes imperative, given their potential impacts on the levels of oncogenes or tumor suppressor genes by modulating the abundance of their target mRNAs. This influence on specific signaling pathways within cancer cells underscores the intricate role of m^6^A readers in the process of carcinogenesis, making it essential to unravel their precise mechanisms and potential therapeutic implications in the context of cancer development.

### M^6^A readers in digestive system cancers

#### Gastric cancer

YTHDF2 exhibits divergent expression patterns and functional roles in gastric cancer (GC). On one hand, it is believed to be significantly upregulated in GC tissues compared to the normal. In vitro assays have demonstrated that silencing YTHDF2 inhibits GC cell proliferation, induces G1 phase arrest, and enhances cell apoptosis [83]. Moreover, the knock-out of YTHDF2 leads to increased expression of Forkhead Box C2 (FOXC2), resulting in the suppression of GC cell proliferation, invasion, and migration [83]. On the other hand, other studies suggest YTHDF2 is downregulated in GC and functions as a tumor suppressor. It is supported by the evidence that up-regulated YTHDF2 is associated with prolonged survival in GC patients [84].

Elevated expression of YTHDF1 has been established as a significant factor in the aggressive progression of GC, as evidenced by in vitro and in vivo studies. This effect is attributed to the m^6^A-dependent facilitation of Frizzled7 (FZD7) translation, a pivotal receptor in the Wnt signaling pathway. Upregulation of YTHDF1 promotes increased FZD7 expression, consequently leading to the hyperactivation of the Wnt/β-Catenin pathway and facilitating the pathogenesis of GC [85]. Additionally, another research indicated that YTHDF1 can facilitate tumorigenesis and GC metastasis by promoting the translation of Ubiquitin Specific Peptidase 14 (USP14) in an m^6^A-mediated manner [86].

#### Pancreatic cancer

YTHDF2 has been found to be upregulated in pancreatic cancer (PC) tissues, and its expression gradually increases with advancing clinical stages. Functionally, YTHDF2 plays a dual role in PC cells, promoting proliferation while inhibiting migration and invasion [87]. In addition, YTHDF2 and ALKBH5 collaborate in PC progression. Loss of ALKBH5 reduces Period Circadian Regulator 1 (PER1) expression in a YTHDF2-dependent manner, promoting tumor migration, invasion, proliferation, and growth. Furthermore, the upregulation of PER1 reactivates the ATM-CHK2-P53/CDC25C signaling pathway, suppressing tumor cell growth [88].

#### Colorectal cancer

YTHDF2 is detected to be downregulated in colorectal cancer (CRC), and it has been found to mediate the degradation of X inactivate-specific transcript (XIST), a long non-coding RNA, which may contribute to accelerated tumor growth and metastasis in CRC [89]. Contrary to previous understanding, recent research suggests a potential carcinogenic role of YTHDF2 in CRC. By downregulating YTHDF2, the stability of m^6^A-modified glycogen synthase kinase 3 beta (GSK3β) mRNA is increased. Consequently, the expression of proteins associated with the Wnt/β-Catenin/Cyclin D1 pathway is inhibited, leading to a suppression of CRC cell proliferation [90]. This emerging evidence highlights the complex and context-dependent nature of YTHDF2's role in CRC, indicating the need for further investigation to fully elucidate its dual functions in the disease.

YTHDF1 is a crucial regulator of tumorigenicity in CRC cells. Its gene copy number positively correlates with mRNA and protein expression levels in CRC. Pertinently, knocking down of YTHDF1 inhibits tumorigenicity in vitro and effectively suppresses murine xenograft tumor growth in vivo, by disrupting the Wnt/β-Catenin pathway [91]. Additionally, YTHDF1 can recognize m^6^A modification on the tumor suppressor gene Ankyrin Repeat and LEM Domain Containing 1 (ANKLE1), which enables YTHDF1 to regulate cell proliferation and enhance genomic stability in CRC carcinogenesis by influencing ANKLE1's transcriptional efficiency [92].

IGF2BPs enhance the aggressiveness of CRC cells and promote vasculogenic mimicry [93, 94]. For example, METTL3-mediated m^6^A modification upregulates circular Ubiquitin Like With PHD And Ring Finger Domains 2 (circUHRF2), subsequently recruiting IGF2BP1 to inhibit the degradation of DEAD-box helicase 27 (DDX27) protein, thereby enhancing CRC stemness and metastasis [93]. In CRC, IGF2BP2 and IGF2BP3 stabilize their transcripts by interacting with m^6^A sites of Ephrin Type-A Receptor 2 (EphA2) and Vascular Endothelial Growth Factor (VEGF), respectively, stimulating vasculogenic mimicry formation. Subsequently, it activates downstream PI3K/AKT/mTOR and MAPK/ERK1/2 signaling pathways to promote cell proliferation, migration, and invasion [94].

#### Hepatocellular carcinoma

Several studies have suggested that YTHDF2 can act as a tumor suppressor in hepatocellular carcinoma (HCC) [95, 96]. Notably, the depletion of YTHDF2, in a hypoxia-inducible factor-2α (HIF-2α)-dependent manner, has been shown to promote HCC cell growth, inflammation, metastasis, and vasculature remodeling. The underlying mechanisms involve YTHDF2 facilitating the decay of interleukin 11 (IL11) and serpin family E member 2 (SERPINE2) mRNAs [97]. Additionally, the expression of YTHDF2 exhibits an inverse relationship with HCC cell proliferation, achieved through the activation of MEK and ERK signaling pathways and the destabilization of the Epidermal Growth Factor Receptor (EGFR) mRNA [95]. Contrary to the above, there is an alternative perspective suggesting that YTHDF2 should be regarded as an oncogene in HCC development, for the deletion of YTHDF2 can decrease POU5F1 translation in an m^6^A-dependent manner, thereby inhibiting the stemness of cancer stem cells [96].

YTHDF1 exerts a pivotal regulatory role in HCC by modulating various cellular processes, including the cell cycle, metabolism, and cell proliferation [98–100]. In recent research, it was revealed that the knock-down of YTHDF1 in HCC cells resulted in substantial inhibition of cell proliferation, migration, and invasion, accompanied by enhanced apoptosis in vitro. In this study, YTHDF1 knock-down has been observed to facilitate the EMT, a pivotal process implicated in the metastatic cascade of liver cancer cells. Furthermore, the activation of the AKT/GSK-3β/β-catenin signaling pathway has been also identified as a potential mechanistic link mediating the observed cellular alterations upon YTHDF1 knock-down [99]. Furthermore, an additional study has elucidated the association between YTHDF1 and HCC cell proliferation. Mechanically, YTHDF1 has been shown to recognize and selectively interact with the m^6^A modification site within the mRNA of Frizzled Class Receptor 5 (FZD5), a key component of the Wnt signaling pathway. This interaction mediates the oncogenic role of YTHDF1 through the activation of the FZD5/Wnt/β-Catenin signaling pathway, thus mediating the HCC cell proliferation [98].

In addition, the IGF2BP family proteins also play a crucial role in regulating HCC proliferation and may significantly influence the tumor progression [101]. For example, the scaffold molecule rtcisE2F has been identified as a key player in mediating the interaction between IGF2BP2 and E2F Transcription Factor 6/3 (E2F6/3) mRNAs, which prevents the decay of E2F6/3 mRNAs and enhances their stability [101]. Interestingly, rtcisE2F also influences the association of E2F6/3 mRNA with another m^6^A reader, YTHDF2, which promotes the decay of E2F6/3 mRNAs. By favoring the binding of IGF2BP2 and inhibiting the association with YTHDF2, rtcisE2F promotes the stability of E2F6/3 mRNAs. This stabilization, in turn, contributes to the activation of the Wnt/β-Catenin pathway and the self-renewal of liver tumor-initiating cells [101].

Studies investigating the cancer-testis-associated long non-coding RNA, namely lnc-CTHCC, have revealed the substantial involvement of IGF2BPs in HCC [102]. These RNA-binding proteins, specifically IGF2BP1 and IGF2BP3, play a crucial role in recognizing the m^6^A modification on lnc-CTHCC, thereby promoting its stability and content within the context of HCC. The upregulated lnc-CTHCC can interact with heterogeneous nuclear ribonucleoprotein K (hnRNPK), thereby forming a complex. This complex then recruits additional Yes1 Associated Transcriptional Regulator (YAP1) promoters, leading to the activation of YAP1 transcription. Consequently, this transcriptional activation contributes to the promotion of hepatocellular carcinogenesis and progression [102].

### Hematological malignancies

Current research on m^6^A modifications in hematopoietic system cancers predominantly focuses on leukemia, specifically acute myeloid leukemia (AML). AML stands out as the most common type of acute leukemia in adults [103]. Despite substantial progress in unraveling the genetic intricacies of AML and strides made in developing personalized treatment approaches, the survival rate for AML patients remains relatively low in comparison to other cancers [104]. In recent years, epigenetic modifications, especially m^6^A, have garnered considerable attention in the exploration of AML therapeutic strategies. Notably, m^6^A readers have been identified as critical players in the pathogenesis of AML, suggesting their potential as novel and effective therapeutic targets for the disease.

As an important m^6^A reader involved in hematopoietic development, YTHDF2 has been discerned to influence tumorigenesis in AML through various pathways. Mice with AML displaying reduced YTHDF2 levels have shown extended survival times, while normal mice with decreased YTHDF2 levels showed an increase in hematopoietic stem cells without any discernible effect on their hematopoietic system functionality. By binding to Tumor Necrosis Factor Receptor 2 (TNFR2), YTHDF2 accelerates the degradation of TNFR2 mRNAs, thus inhibiting the TNF signaling pathway and participating in AML progression [105]. Simultaneously, YTHDF2 identifies m^6^A modification sites in pre-miR-126 and collaborates with the pre-miRNA regulator Argonaute2 (AGO2), facilitating the conversion of precursor-miR-126 to mature miR-126. This process upregulates the expression of miR-126-3p, a known AML oncomiR, which plays a promotive role in tumorigenesis. Therapies targeting the YTHDF2/miR-126 axis hold considerable therapeutic potential [106]. Additionally, in the t(8;21) AML subtype, YTHDF2 is observed to have elevated expression levels, which are correlated with a higher risk of relapse. Subsequent studies have indicated that YTHDF2 is a downstream target of the AML1/ETO-HIF1α pathway. This axis may promote cellular proliferation in t(8;21) AML by modulating global m^6^A modification [107].

Although being identified as a non-essential factor for normal hematopoiesis in mice, YTHDF1 is imperative for the progression of AML [108]. Both in vivo and in vitro knock-outs of YTHDF1 in AML cells have demonstrated a significant diminish in their self-renewal, proliferation, and pathogenic capacities. Subsequent investigations revealed that YTHDF1 promotes the translation of Cyclin E2 in an m^6^A-dependent manner, thereby influencing the normal cell cycle [108]. Tegaserod, a potential inhibitor that functions by disrupting the interaction between YTHDF1 and m^6^A-modified mRNAs, represents a promising therapeutic agent. Clinical trials have shown that Tegaserod can reduce the in vitro viability of AML cells derived from patients and prolong their lifespan [108].

Similar to YTHDF1, while not significant for hematopoiesis development, YTHDC1 has also been unequivocally demonstrated to be indispensable for AML cell survival, differentiation, and leukemogenesis, advancing the progression of leukemia through various mechanisms [109, 110]. Nuclear YTHDC1-m^6^A condensates (nYACs), resulting from liquid–liquid phase separation of YTHDC1 and m^6^A, are observed to be more prevalent in AML cells than in normal cells. These nYAC condensates are harnessed to sustain AML cell survival and an undifferentiated state, which are critical for the persistence of leukemia [109]. A recent publication highlighted the role of YTHDC1 in binding to the HOXB-AS3 complex and modulating its expression, which is strongly associated with poor prognosis in AML patients. Over-expression of either YTHDC1 or HOXB-AS3 can amplify the proliferation of leukemia stem cells (LSCs), and impair their apoptosis. This leads to an increased presence of LSCs in the blood and bone marrow of AML mice, thereby sustaining leukemia progression [110]. Furthermore, the RBFOX2-MTC-YTHDC1 transcriptional regulatory axis, by recruiting Polycomb Repressive Complex 2 (PRC2), facilitates the interaction between YTHDC1 and PRC2, consequently suppressing chromatin accessibility in bone marrow cells. When YTHDC1 is recruited to the gene promoter regions, it can destabilize m^6^A-modified promoter-associated noncoding RNA 1 (paRNA1) and mediate H3K27me3 to repress normal RNA transcription [53].

Overexpressed in AML, IGF2BPs are crucial in the maintenance of the disease state.YBX1, a protein that is integral to AML, interacts with IGF2BPs, thereby stabilizing tumor-related genes that contain m^6^A mRNAs, such as V-Myc Avian Myelocytomatosis Viral Oncogene Homolog (MYC) and BCL2 Apoptosis Regulator (BCL2) [111]. IGF2BP1 has been identified as promoting the expression of oncogenic factors by counteracting miRNA-mediated suppression in cancer [112]. Research demonstrates that the tumorigenicity of leukemia cells can be reduced by inhibiting the expression of IGF2BP1 genetically or chemically. This interaction not merely promotes bone marrow differentiation but also enhances the susceptibility of leukemia cells to chemotherapeutic agents [113]. IGF2BP2, by targeting genes involved in glutamine (Gln) metabolism, such as Glutamic–Pyruvic Transaminase 2 (GPT2), Solute Carrier Family 1 Member 5 (SLC1A5), and MYC, supports the self-renewal of leukemia stem cells and progenitor cells, thereby facilitating AML progression. This regulatory process can be inhibited by the small molecule drug CWI1-2 [114]. Additionally, IGF2BP3 accelerates the progression of AML by enhancing the Regulator of Chromosome Condensation 2 (RCC2) stability [115]. However, the detailed mechanisms of IGF2BP3, as well as other m^6^A readers in AML, remain elusive.

Research regarding the role of m^6^A readers in other leukemias is still limited. Only IGF2BP3 is overexpressed in a leukemia subtype (MLL-rearranged B-ALL) with a poor prognosis. Tumor cells with knock-out IGF2BP3 displayed increased apoptosis and reduced proliferation [116]. To conclude, the research on m^6^A readers in hematopoietic system-related cancers is still lacking and needs further studies.

### Neurological-related cancers

In glioblastoma stem cells, YTHDF2 plays a role in maintaining its oncogenic phenotype by stabilizing MYC and Vascular Endothelial Growth Factor (VEGF) [117]. Moreover, in glioblastoma, YTHDF2 undergoes phosphorylation at serine 39 and threonine 381 through EGFR/SRC/ERK signaling pathway. This phosphorylation stabilizes the YTHDF2 protein, which in turn facilitates the decay of Nuclear Receptor Subfamily 1 Group H Member 3 (NR1H3, also named LXR-A) and HIVEP Zinc Finger 2 (HIVEP2) mRNAs. The degradation of these specific mRNAs leads to cholesterol dysregulation, cell proliferation, invasion, and tumorigenesis of glioblastoma [118].

YTHDC1, identified as a crucial factor in glioblastoma, can modulate glioblastoma development through interactions with specific mRNA molecules [119]. Specifically, YTHDC1 binds to the start codon region of SRSF3, SRSF6, and SRSF11 mRNAs, triggering the decrease of these SRSF protein levels via the m^6^A-dependent nonsense-mediated mRNA decay pathway. This process affects target mRNA alternative splicing, including BCL-X and nuclear receptor corepressor 2 (NCOR2), consequently contributing to the glioblastoma phenotype [119].

Neuroblastoma patients with elevated IGF2BP3 expression tend to have undifferentiated histology, advanced stages, and an unfavorable prognosis. Moreover, IGF2BP3 expression was observed to be decreased following retinoid acid treatment, and its knock-down resulted in reduced invasion ability of neuroblastoma cells [120]. These findings imply a potential involvement of IGF2BP3 in promoting the undifferentiated phenotype and invasive behavior of neuroblastoma, emphasizing its prognostic significance and potential as a therapeutic target. Additionally, in a recent study, IGF2BP3 has been identified as a biomarker that can be used in the initial diagnosis of meningioma [121].

### Respiratory system cancers

YTHDF2, a prominent player in the regulation of post-transcriptional events, has emerged as a key modulator of YAP mRNA degradation in the context of non-small cell lung cancer (NSCLC) [122, 123]. This regulatory process is intricately governed by the m^6^A modification and interaction with the AGO2 system, which is closely associated with the functionality and maturation of miRNAs. By facilitating the targeted breakdown of YAP mRNA, YTHDF2 effectively influences important cellular processes such as growth, angiogenesis, and metastasis in NSCLC cells. Interestingly, YTHDF1 and YTHDF2 were found to competitively interact with YTHDF3 during the regulation of YAP mRNA levels, and this interaction occurs independently of the m^6^A modification [122]. Moreover, recent studies have demonstrated the regulatory role of YTHDF2 in modulating the expression of Axin1, a critical negative regulator of the Wnt/β-Catenin pathway. YTHDF2 mediates this regulation by targeting AXIN1 mRNA, leading to a reduction in its expression through the acceleration of mRNA decay. Consequently, this molecular mechanism contributes to promoting lung adenocarcinoma (LUAD) progression [123].

YTHDF1 exhibits elevated expression in NSCLC cells and exerts suppressive effects on apoptosis, while promoting proliferation and cellular aggressiveness [122, 124]. Recent research highlights the crucial role of YTHDF1 in driving the progression of NSCLC through its mediation of the Hippo pathway via interaction with its effector, YAP. Specifically, YTHDF1 recognizes the m^6^A modification present in YAP mRNAs and forms a complex with eIF3a, thereby enhancing the translational efficiency of YAP mRNAs. This regulatory mechanism has been shown to exert profound effects on cellular growth, invasion, and EMT in NSCLC cells, as substantiated by comprehensive investigations conducted both in vitro and in vivo [122]. In addition, another investigation has demonstrated the m^6^A-dependent role of YTHDF1 in regulating the translation efficiency of cyclin-dependent kinase 2 (CDK2), CDK4, and cyclin D1 (CCND1), thereby exerting control of NSCLC cell proliferation and xenograft tumor formation [124].

IGF2BPs enhance the proliferation and migration of NSCLC cells. Specifically, IGF2BP1 stabilizes Salt-Inducible Kinase 2 (SIK2) mRNA through m^6^A modification, leading to an upregulation of SIK2 levels [125]. Overexpression of SIK2 suppresses the Hippo/YAP pathway, promoting cell proliferation, migration, invasion, and inhibiting apoptosis, ultimately facilitating the progression of NSCLC [125]. In addition, stimulated by the promoter Striatin Interacting Protein 2 (STRIP2), IGF2BP3 transcription is upregulated. Subsequently, the STRIP2-IGF2BP3 axis recognizes m^6^A modification on Transmembrane BAX Inhibitor Motif Containing 6(TMBIM6) mRNA and enhances the stability of TMBIM6, ultimately promoting NSCLC cell proliferation, migration, and invasion. High co-expression of STRIP2, IGF2BP3, and TMBIM6 in NSCLC patients is associated with a shorter survival rate [126].

### Cancers in other systems

#### Bladder cancer

YTHDF2, identified as an oncogene in bladder cancer (BC), exhibits upregulation in BC patients. Notably, according to the analysis of MeRIP and RIP, it has been revealed that the METTL3/YTHDF2 axis plays a critical role in the degradation of the mRNAs of tumor suppressors including SET Domain Containing 7 (SETD7) and KLF4, which potentially contributes to the progression of BC [127].

YTHDF1 has been demonstrated to play a vital role in promoting BC progression [128, 129]. One underlying mechanism involves the interaction of YTHDF1 with the m^6^A-modified 3′UTR of CUB Domain Containing Protein 1 (CDCP1) mRNA, resulting in enhanced translation of CDCP1. The upregulated expression of CDCP1, in turn, interacts with the oncogenic Ras/ERK pathway, leading to the promotion of growth, migration, and invasion of BC cells [128]. Similarly, YTHDF1/YTHDF3 has been implicated in increasing the translational efficiency of Integrin Subunit Alpha 1 (ITGA), one of the genes that are defined as “tumor differentiation signature”, through the recognition of the m^6^A site present on its 3′UTR. This enhanced translation leads to an upregulation of ITGA expression, which in turn contributes to the promotion of BC cell growth [129].

Additionally, it has been observed that IGF2BP3 exhibits elevated expression levels in BC tumor samples. Importantly, this upregulation of IGF2BP3 has been associated with the regulation of both total and membrane-bound expression levels of programmed cell death ligand 1 (PD-L1), which has been established to be associated with immune suppression and tumor progression [130].

#### Prostate cancer

Similarly, YTHDF2 has been identified to be upregulated in prostate cancer (PCa), while its role in disease progression is mediated through a distinct mechanism [131]. Specifically, YTHDF2 mediates the m^6^A-dependent mRNA degradation of tumor suppressors, such as phospholysine phosphohistidine inorganic pyrophosphate phosphatase (LHPP) and NK3 Homeobox 1 (NKX3-1). This regulatory process ultimately contributes to the modulation of AKT phosphorylation and subsequent tumor progression in PCa [131].

YTHDF1 has been found to play an oncogenic role and upregulated in the PCa tissue [132]. Specifically, dysregulation of the ELK1 can directly interact with the promoter region of YTHDF1 and lead to the activation of its transcription. Subsequently, YTHDF1 can exert its tumorigenic effects by targeting Polo Like Kinase 1 (PLK1) in an m^6^A-dependent manner, which facilitates the hyperactivation of the PI3K/AKT signaling pathway, thereby promoting PCa tumorigenesis and metastasis both in vitro and in vivo [132].

YTHDC1 has demonstrated a novel nuclear role in PCa. By interacting with metadherin, a key contributor to tumorigenesis, YTHDC1 exerts a repressive effect on the exon inclusion of tumor-related mRNA, such as the CD44v5-luc minigene [133]. Consequently, YTHDC1's regulatory function impacts the processes of carcinogenesis and cancer advancement in PCa.

Recent research has also revealed that IGF2BP2 can act as an oncogene in PCa. Functional studies have demonstrated a significant inhibition of PCa cell invasion, migration, and proliferation upon knock-down of IGF2BP2 in vitro [134]. The mechanistic basis of IGF2BP2's role in PCa involves METTL3-mediated m^6^A modification of Prostate Cancer Associated Transcript 6 (PCAT6), leading to the upregulation of PCAT6 in an IGF2BP2-dependent manner [134]. Additionally, IGF2BP2 plays a pivotal role in stabilizing Insulin-Like Growth Factor 1 Receptor (IGF1R) mRNA through the formation of a PCAT6/IGF2BP2/IGF1R RNA–protein complex. This intricate complex serves to enhance IGF1R expression, thereby further fueling the progression of PCa [134].

#### Breast cancer

The deficiency of YTHDF1 accelerates the growth of breast cancer cells both in vitro and in vivo, suggesting its possible role as an oncogenic factor in breast cancer [135]. According to a recent study, YTHDF1 facilitates breast cancer cells to proliferate and invade, boosting tumorigenicity and metastasis through promoting glycolysis. The expression of YTHDF1 and miR-16-5p (a miRNA) are post-transcriptionally inhibited and promoted, respectively, by tumor hypoxia, which also induces Hypoxia Inducible Factor 1 (HIF1) through transcription. PKM2 is then upregulated, which promotes tumor glycolysis and ultimately increases the tumorigenic and metastatic potential of breast cancer cells [136]. Additionally, it has been demonstrated that Forkhead Box M1 (FOXM1) is a target of YTHDF1. By identifying and binding m^6^A-modified FOXM1 mRNA and speeding up FOXM1 translation, YTHDF1 aids in the spread of breast cancer. The overexpression of FOXM1 in breast cancer cells partially offset the tumor suppressor impact of YTHDF1 silencing [137].

Many m^6^A readers have been proven to be associated with distant metastasis of breast cancer and are considered promising prognostic biomarkers and therapeutic targets for breast cancer, here IGF2BP1 and YTHDF3 are examples [138–140]. IGF2BP1 is stabilized by deubiquitination mediated by Ubiquitin-Specific Peptidase 10 (USP10), resulting in its elevated expression in breast cancer [140]. IGF2BP1 directly recognizes, binds to, and enhances the stability of the m^6^A site on Carnitine Palmitoyltransferase 1A (CPT1A) mRNA, ultimately mediating IGF2BP1-induced metastasis [138]. Through its mechanism, YTHDF3 increases the translation of m6A-enriched transcripts associated with cerebral metastasis of breast cancer, such as ST6 N-Acetylgalactosaminide Alpha-2,6-Sialyltransferase 5 (ST6GALNAC5), Gap Junction Protein Alpha 1 (GJA1), and EGFR. This results in various outcomes, such as increased interaction between cancer cells, brain endothelial cells, and astrocytes, heightened blood–brain barrier spillover, angiogenesis, and tumor cell growth [140].

#### Cervical cancer

YTHDF1 occupies a crucial role in modulating cancer metabolism and promoting tumorigenesis in cervical cancer cells. It is recruited by METTL3 to enhance the stability of hexokinase 2 (HK2), exerting an oncogenic factor by promoting the Warburg effect (aerobic glycolysis) in cervical cancer. Since the Warburg effect is known as a typical hallmark of cancer metabolism, its increase is associated with tumorigenesis [141]. Additional studies have analyzed RIP-seq, meRIP-seq, and ribosome profiling sequencing (Ribo-seq) data after YTHDF1 knock-down and found that RAN Binding Protein 2 (RANBP2) is a key target of YTHDF1 in cervical cancer cells. YTHDF1 regulates the translation of RANBP2 in an m^6^A-dependent manner, but not its mRNA expression, thereby affecting cervical cancer cell growth, migration, and invasion [142].

IGF2BP2 promotes cervical cancer fate by specifically binding to circRNAs and thereby regulating circRNAs [143]. circARHGAP12, a novel circRNA, was found to be upregulated in cervical cancer tissues and adjacent normal tissues by circRNA high-throughput sequencing. Mechanistically, IGF2BP2 recognizes the m^6^A site in circARHGAP12 and simultaneously enhances its enrichment, thereby promoting the proliferation and migration of cervical cancer [143].

#### Merkel cell carcinoma

A recent study has shown that YTHDF1 plays an oncogenic role through m^6^A machinery in the tumorigenesis of Merkel Cell Carcinoma (MCC) [144]. M^6^A modification sites have been identified on the Merkel cell polyomavirus (MCPyV) sequence, indicating their potential involvement in MCC pathogenesis. Increased expression of YTHDF1 has been shown to activate cap-dependent translation by working with eIF3A and eIF3B. This coordinated translational initiation process may contribute to the acquisition of the highly tumorigenic cell characteristics in MCC [144].

#### Ocular melanoma

The role of YTHDF3 In ocular melanoma has been investigated, revealing its essential contribution to oncogenic capacity and tumor propagation [145]. High expression levels of YTHDF3 have been observed in melanoma cancer stem cells (CSC), and the knock-down of YTHDF3 has been found to significantly inhibit tumor proliferation and migration, emphasizing its critical involvement in these processes [145]. Mechanistically, YTHDF3 has been found to facilitate the translation of Catenin Beta 1 (CTNNB1), a driver gene implicated in melanoma genesis, via an m^6^A-dependent mechanism. CTNNB1 activation, through the Wnt/β-Catenin pathway, subsequently contributes to melanoma progression and metastasis [145].

In summary, m^6^A readers play key roles in diverse systems and biological processes. Mis-regulation of these m^6^A readers contributes to different types of cancers, not only highlighting the importance of m^6^A-mediated regulation but also implying the m^6^A readers may serve as drug targets for cancer therapeutics [146] (Fig. 3).

## Roles of m^6^A readers in other noncancer diseases

### Infectious diseases

A recent study has revealed that YTHDF1 may have an alleviatory effect on sepsis, which results from a dysfunction in the host's response to infection [147]. Sepsis is closely related to pyroptosis, a caspase-1-dependent programmed cell death [147]. Mechanistically, YTHDF1 has been shown to enhance the translation of WW domain-containing E3 ubiquitin protein ligase 1 (WWP1) by relying on an m^6^A-dependent process. Consequently, the increased expression of WWP1 leads to the ubiquitination of another protein called NOD-like receptor family pyrin domain-containing 3 (NLRP3), while also inhibiting caspase-1-dependent pyroptosis. This regulatory cascade mediated by YTHDF1 and WWP1 exerts inhibitory effects on inflammation and immune cell apoptosis, thereby contributing to the alleviation of sepsis symptoms [147].

Additionally, YTHDFs were found to play a vital role in inhibiting HIV-1 infection by disturbing viral reverse transcription [148]. This inhibitory effect is achieved through the interaction between YTHDF proteins and m^6^A methylation marks present on the genomic RNAs of HIV-1. The engagement of YTHDFs with these m^6^A modifications triggers the degradation of viral RNAs, thereby impeding the process of reverse transcription and subsequent viral gene expression [148]. Consequently, YTHDF proteins act as negative regulators of post-entry HIV-1 infection, providing a potential avenue for therapeutic interventions targeting the m^6^A-modified viral RNA [148].

### Cardiovascular diseases

Recent research has revealed the essential role of YTHDC1 in dilated cardiomyopathy (DCM), which is caused by left ventricular dilatation and contractile dysfunction [149]. Deficiency of YTHDC1 has been shown to lead to noticeable enlargement of the left ventricular chamber, severe systolic dysfunction, reduced cardiomyocyte contractility, and disrupted sarcomere organization [149]. Furthermore, through the utilization of multiple high-throughput sequencing technologies, it has been discovered that Titin mRNAs, which undergo m^6^A modification, are a potential downstream target of YTHDC1. It has been observed that the depletion of YTHDC1 leads to abnormal splicing patterns of Titin, further implicating YTHDC1 in the regulation of cardiac function [149].

### Lesions associated with diabetes

Diabetic foot ulcers are characterized by impaired wound healing, and recent research has shown the potential involvement of YTHDC, specifically within diabetic keratinocytes. It was discovered that YTHDC1, in collaboration with ELAV-like RNA binding protein 1 (ELAVL1)/HuR, regulates the stability of SQSTM1 nuclear mRNA in diabetic keratinocytes, which affects the autophagy and the wound healing rate of diabetic skin [150].

Additionally, m^6^A readers have been identified to modulate glucose-stimulated insulin secretion during the different phases of diabetes development. Specifically, it has been observed that the presence of the IGF2BP2 variant leads to a significant decrease in insulin secretion during the first phase, which corresponds to the initial rapid release of insulin in response to glucose stimulation [151]. Moreover, it has been found that the role of IGF2BP2 in diabetic nephropathy (DN) depends on interlinked communication with several other genes, miRNAs, and lncRNAs [152]. Specifically, one critical component involved in DN pathogenesis is Laminin-β2 (LAMB2), which plays a crucial role in determining the permeability of the glomerular basement membrane. IGF2BP2 directly targets lamb2 mRNA within the actin cytoskeleton, allowing it to regulate LAMB2 expression [152]. In DN, elevated glucose concentrations have been observed to increase the levels of let-7b, a specific miRNA. Let-7b suppresses the expression of a transcription factor, High Mobility Group Protein 2 (HMGA2). Consequently, the downregulation of HMGA2 leads to a subsequent reduction in the expression levels of IGF2BP2 and LAMB2 [152].

## Perspectives

In conclusion, this review emphasizes the critical role of m^6^A readers in governing gene expression. Throughout normal early developmental processes, m^6^A readers exhibit significant influence over stem cell fate by targeting specific transcripts. Additionally, dysregulated levels of m^6^A readers in cancers disrupt the mRNA levels of their target genes, thereby perturbing signaling pathways such as Wnt/β-Catenin, ultimately impacting cancer cell proliferation, invasion, and metabolism.

However, the mechanisms underlying how m^6^A readers select target transcripts with m^6^A modifications remain elusive. For example, there is controversy regarding whether different YTHDFs respectively recognize dissimilar m^6^A sites in the cytoplasm. Initially, the YTHDF family was believed to have distinct functions: YTHDF1 enhancing mRNA translation, YTHDF2 promoting mRNA degradation, and YTHDF3 modulating both translation and degradation [13, 32]. However, subsequent studies have revealed that YTHDF proteins do not regulate HeLa cell translation, and during mRNA degradation, they bind to the same m^6^A-modified mRNAs rather than distinct ones [153]. Furthermore, current research on m^6^A readers predominantly focuses on YTH family proteins and a few members of the IGF2BP family, while other types of readers seem to receive insufficient attention. Therefore, further exploration is required to elucidate the specific mechanisms underlying the actions of other readers. With an increasing number of studies uncovering the close association between m^6^A modification and cancers, several compelling questions arise: How do several readers coordinate transcriptional regulation at the single-cell level? Why do certain m^6^A readers exhibit opposing effects in different types of cancers? And through which mechanisms do these readers selectively modulate the regulation of downstream transcripts in distinct cellular contexts?

Meanwhile, some m^6^A readers possess additional biological functions beyond recognizing m^6^A sites. For instance, IGF2BP proteins stabilize Insulin-like Growth Factor 2 (IGF2) mRNA, enhancing its role in the IGF signaling pathway, thereby influencing glucose metabolism [154, 155]. Therefore, whether m^6^A reader proteins regulate certain diseases in an m^6^A-dependent manner remains to be elucidated. For example, IGF2BP2 may influence glucose homeostasis and the progression of diabetes by modulating laminin-β2 mRNA levels, but whether this regulation occurs through recognizing m^6^A modifications requires further verification [152].

It is crucial to translate the foundational research into practical clinical applications. Recently, it has been suggested that a shift in focus toward specific RNA transcripts and m^6^A sites, as opposed to global m^6^A levels, could offer valuable insights into their potential as cancer biomarkers. With advancements in m^6^A-seq methods like m^6^A-Selective Allyl Chemical labeling and sequencing (m^6^A-SAC-seq) and evolved TadA-assisted N6-methyladenosine sequencing (eTAM-seq), we can now use clinical samples to create detailed maps of m^6^A modifications within transcriptomes with single-base resolution [77, 156]. These maps hold promise in identifying m^6^A features on specific mRNA sites, making them potential biomarkers for cancer diagnosis, classification, prognosis, risk assessment, and treatment decisions. Furthermore, utilizing the power of artificial intelligence and machine learning may greatly enhance our ability to analyze large-scale datasets of m^6^A modification. This has the potential to transform our understanding of how m^6^A readers interact with their downstream targets and could revolutionize disease diagnosis, particularly in cancers. Additionally, there is growing interest in using pharmacological agents to target dysregulated m^6^A regulators as a promising therapeutic approach. In addition to the investigation into tegaserod's targeting of YTHDF1 for AML treatment, recent research has unveiled Salvianolic acid C (SAC), a principal constituent of the traditional Chinese medicine Danshen, as a promising YTHDF1 inhibitor. This discovery presents a potential way for alleviating symptoms associated with Fragile X syndrome [157]. Although only a few small molecule drugs targeting m^6^A readers have entered clinical trials, developing highly effective and selective inhibitors with favorable therapeutic profiles remains an exciting area for further exploration. In view of the different m^6^A readers in development and diseases, deeper studies will provide new insights into how the epigenetic information is encoded by m^6^A modification and how to administer medications targeting m^6^A readers for various diseases.

## Data Availability

Not applicable.
